# Afghan hurdles: from signing the pledge in 2012 to hand hygiene implementation in 2014

**DOI:** 10.1186/2047-2994-4-S1-P149

**Published:** 2015-06-16

**Authors:** JG Böttrich, Z Tokhi, B Roth, D Pittet, K-W Stahl

**Affiliations:** 1 Waisenmedizin PACEM, Switzerland, Wangen bei Olten, Switzerland; 2 Pharmaceutical Faculty, Balkh University, Mazar-e-Sharif, Afghanistan; 3 Waisenmedizin PACEM, Wangen bei Olten, Switzerland; 4 Infection Control, Hôpitaux Universitaires de Genève, Genève, Switzerland; 5 Waisenmedizin e.V., Freiburg / Breisgau, Germany

## Introduction

Cutaneous Leishmania (CL) skin defects lasting for >6 weeks^1^ similar to chronic wounds of the elderly in developed countries are prevalent in Afghanistan^2^ in uncovered body parts mostly of young children after their first contact with the parasite through a sand-fly bite. From two phase II CL trials^3,4^ it appears that clean CL wound management is crucial for rapid healing.

## Objectives

As alcohol-based hand-rubs (ABHR) were not available in Afghanistan^5^ in 2012 when signing the WHO pledge^6^ to reduce the burden^7^ of the CL disease, we wanted to foster the local low-price ABHR production by involving the Pharmaceutical Faculty on the new Campus (PharmFac).

## Methods

In the absence of PharmFac labs, we used the lab of the renovated leishmania centre of the Balkh Civil Hospital, which two of us had (DP & KWS) audited in 2012. We trained the students on the job of producing ABHR according to the WHO guide using the WHO starter kit^8^. To prepare 3 batches of 100 ml ABHR bottles we had to buy the ingredients of WHO formula 1 on the Mazar bazaar.

## Results

The students, who participated in the daily leishmania wound patients’ consultations, were highly motivated, when they understood the importance of hand-hygiene in this field. They were enthusiastic about their first opportunity of pharmaceutical bench work. They were hugely disappointed, when the later quality control at the HUG Pharmacy in Geneva revealed that two flasks of “Ethanol absolut” had been adulterated with methanol, up to 50 and 80% respectively, and that they had to destroy all 150 ABHR flasks on the spot. In 2014 ABHR leftover stocks of the US Army had engulfed the bazaar of Mazar at 1/2.6 USD per litre (October 2014/February 2015). The temporary economic ABHR availability might explain the hand hygiene awareness and practice we observed in the Balkh Civil Hospital in contrast to what we had experienced in 2012.

## Conclusion

Economic ingredients of guaranteed quality constitute the bottleneck for ABHR production in poor countries, not the motivation of pharmaceutical students. Those, who feel concerned, are invited to discuss such issues with us.

## Disclosure of interest

J. G. Böttrich Employee of: B. Braun Melsungen AG, Z. Tokhi: None declared, B. Roth: None declared, D. Pittet: None declared, K.-W. Stahl: None declared.

